# Cutting Through the Debate: Surgical Resection Versus Surveillance-Based Approach for Kikuchi SM2/3 Colorectal Polyps

**DOI:** 10.7759/cureus.97774

**Published:** 2025-11-25

**Authors:** Izna Najam Syed, Noem Najam Syed, Lin Alkhiami, Bryony Bowman, Syed Umer Hannan, Deepak Singh-Ranger

**Affiliations:** 1 General Surgery, The Royal Wolverhampton NHS Trust, Wolverhampton, GBR; 2 General Surgery, Dow University of Health Sciences, Karachi, PAK; 3 Internal Medicine, Hampshire Hospitals NHS Foundation Trust, Winchester, GBR; 4 Haematology, Hampshire Hospitals NHS Foundation Trust, Basingstoke, GBR; 5 General Surgery, Alrayan Colleges, Medina, SAU; 6 General and Colorectal Surgery, New Cross Hospital, Wolverhampton, GBR

**Keywords:** bowel cancer screening, colon cancer surveillance, colonoscopy surveillance, colorectal cancer, kikuchi classification, prophylactic colectomy, sessile polyps

## Abstract

Background

Malignant colorectal polyps are cancer lesions that invade the submucosa but do not extend into the muscularis propria. The Kikuchi system of malignant polyp classification is used for sessile polyps and describes the depth of cancer invasion into the submucosa (SM1-3). With deeper submucosal invasions (SM2 and SM3), the risk of lymph node involvement is higher. Therefore, these patients should be considered for prophylactic surgical resection. Despite this, we have noted a greater inclination toward a surveillance-based approach for Kikuchi SM2/3 malignant colorectal polyps due to the absence of clear guidelines regarding the management of sessile polyps. This study aimed to compare the prognostic benefits of a surveillance-based and surgical approach in patients with Kikuchi SM2/3 polyps by evaluating the rate of recurrence and case fatality.

Methodology

This retrospective cohort study evaluated the oncological outcomes of a surveillance-based approach versus prophylactic colectomy in Kikuchi SM2 and SM3 polyps identified in individuals participating in the UK’s National Bowel Cancer Screening Programme. The study was conducted at New Cross Hospital in Wolverhampton, United Kingdom, over a 14-year period, from February 2009 to February 2023. Patients with pedunculated polyps, lower Kikuchi classification, and histologically unassessable polyp classification were excluded. Data analysis was performed using SPSS Statistics for Windows, Version 24.0 (IBM Corp., Armonk, NY, USA).

Results

This study included 70 sessile polyps with a Kikuchi classification of SM2. Of the 70 cases studied, 23 opted for surgical resection, while 47 chose surveillance-based management. No significant differences in baseline demographics (age and gender) were observed between the groups. Oncological outcomes revealed that the degree of endoscopic and biochemical recurrence was significantly higher in the surveillance group versus the surgical group. Cancer-related mortality was statistically significantly lower in the surgical group. Radiological recurrence was lower in the surgical group but not statistically significant. Metastatic lymph node involvement in the surgical group was significant.

Conclusions

This study suggests that the surgical management of Kikuchi SM2 malignant colorectal polyps offers better prognostic outcomes, with lower rates of cancer-related mortality and fewer patients developing clinical recurrence. Surgeons may consider these practices based on the study findings.

## Introduction

Colorectal cancer (CRC) is among the most common cancers globally, representing a significant public health burden in both high- and middle-income countries [1]. Data from the GLOBOCAN 2021 database indicate that CRC is the third most fatal and fourth most frequently diagnosed cancer worldwide, with over 1.8 million new cases and 880,000 deaths annually [2]. As the global burden of CRC continues to show an upward trajectory, the need for precise, evidence-based approaches to early detection and management is even more evident, particularly for early invasive lesions such as malignant colorectal polyps.

Malignant colorectal polyps are histologically defined as lesions characterized by invasion of neoplastic cells into the submucosa without extension into the muscularis propria [1]. These lesions can arise from one of two pathways: adenomatous or serrated, representing an intermediate stage between non-invasive adenomas and overt colorectal cancers [2]. Accurate assessment of the depth of invasion is crucial for determining risk stratification and ultimately appropriate management strategies by weighing the risk of disease progression against the morbidity associated with treatment [3]. One of the key determinants of the need for intervention is the depth of mucosal invasion, which serves as an indicator for the probability of lymphovascular involvement or nodal metastasis.

The classification systems employed to determine the depth of invasion are the Haggitt classification for pedunculated polyps and the Kikuchi classification for sessile polyps. The Kikuchi classification system stratifies sessile polyps based on submucosal invasion depth into three levels: SM1, SM2, and SM3. Deeper invasions, specifically SM2 (invasion into the middle third) and SM3 (invasion into the lower third of the submucosa), are associated with increased risks of lymph node metastasis, with reported rates of metastasis being 8% and 23%, respectively. Consequently, current guidelines often recommend prophylactic surgical resection for patients with these classifications to mitigate the risk of metastatic spread, which is not achievable with endoscopic polypectomy alone [1].

However, recent clinical practice reveals a growing inclination toward surveillance-based management for Kikuchi SM2 and SM3 malignant colorectal polyps. Some studies suggest that certain patients may safely undergo surveillance without immediate surgical intervention, provided there are favorable histological features (such as clear resection margins, moderate-to-well differentiation) and no evidence of lymph node involvement. Conversely, other research emphasizes the potential risks of conservative management, advocating for surgical resection to ensure complete removal and accurate staging [4].

This trend is also largely attributed to (and complicated by) the absence of clear, standardized guidelines addressing the management of sessile cancerous polyps, leading to variability in treatment approaches [5]. A recent study by Park et al. (2024) highlighted this variability and emphasized the need to formulate clearer and more precise criteria to guide the decision-making for the management of early invasive CRC [6]. Moreover, the decision between surveillance and surgery is further complicated by variations in clinical practice and patient outcomes. Factors such as tumor differentiation, patient age, and gender have been shown to influence management strategies. For instance, patients with well or moderately differentiated tumors are less likely to undergo surgery compared to those with poorly differentiated tumors. Additionally, younger patients and males may have different management outcomes, highlighting the need for personalized treatment plans [7].

Several retrospective cohort studies have begun to clarify which patients may benefit from surveillance. These studies suggest that individuals with SM2 or SM3 lesions who meet favorable pathological criteria may be safely monitored, reducing unnecessary risks associated with surgical interventions. Conversely, other research supports the traditional approach of surgical resection, citing concerns about underestimation of lymphovascular invasion and nodal spread on initial biopsy [8-10].

The implications of overtreatment must also be considered. Surgical resection of colorectal segments can be associated with complications such as bleeding, infection, anastomotic leakage, and long-term changes in bowel function. These risks are particularly concerning in elderly or frail patients, where postoperative morbidity and reduced quality of life must be weighed against uncertain oncological benefit [11].

The management of malignant colorectal polyps, particularly those classified as Kikuchi SM2 and SM3, presents a nuanced clinical challenge that necessitates a careful balance between surgical intervention and surveillance strategies. These lesions are associated with a higher risk of lymph node metastasis compared with more superficial polyps, prompting ongoing debate regarding whether immediate surgical resection or structured surveillance offers the most appropriate balance of oncological safety and procedural risk. The clinical rationale for this study stems from the increasing use of endoscopic mucosal resection (EMR) in routine practice, resulting in more SM2 and SM3 lesions being detected incidentally after endoscopic removal. Consequently, clinicians are frequently faced with difficult decisions regarding the need for completion surgery versus close follow-up.

This study aims to address this knowledge gap by directly comparing outcomes between surveillance-based management and surgical intervention in patients with Kikuchi SM2 and SM3 malignant colorectal polyps. Specifically, we evaluate recurrence rates, encompassing endoscopic, radiological, and biochemical indicators, as well as cancer-related mortality. Through this comparison, we seek to test the hypothesis that, in selected patients, structured surveillance may offer comparable oncological outcomes to surgery while avoiding its associated risks. Generating robust data in this area is essential to informing evidence-based decision-making and refining management protocols for these high-risk lesions.

This article was previously presented as a poster abstract at the European Society of Coloproctology (ESCP)’s 19th Scientific and Annual Conference on September 25-27, 2024.

## Materials and methods

Study design, duration, and population

This retrospective cohort study was conducted over a 14-year period, from February 2009 to February 2023, at New Cross Hospital in Wolverhampton, United Kingdom. It focused on patients who underwent colonoscopies as part of the Bowel Cancer Screening Programme, as well as those who underwent colonoscopies for suspected bowel cancer outside of the screening program. The study specifically examined patients diagnosed with sessile colorectal polyps, and the data were collected retrospectively from clinical records. All polyps included in the study were removed using EMR; no lesions were resected via endoscopic submucosal dissection.

Inclusion and exclusion criteria

Our study included non-randomized patients aged 30-93 years who had an endoscopic diagnosis of Kikuchi SM2 and SM3 sessile malignant colorectal polyps. This included patients who underwent colonoscopies as part of the UK’s national Bowel Cancer Screening Programme, as well as patients who were referred for endoscopic investigation for symptoms suspicious for colorectal cancer, over the 14-year period. Both surveillance-based management and prophylactic surgical approaches were included. SM2 and SM3 lesions were selected for analysis because these deeper submucosal invasive polyps carry a higher risk of lymph node metastasis and represent the group for which the decision between surveillance and surgery remains most clinically uncertain. Patients with pedunculated polyps, lower Kikuchi classification, and histologically unassessable polyp classification were excluded.

Recruitment and consent

As part of the UK’s national Bowel Cancer Screening Programme, all individuals aged between 54 and 74 years received an invitation and a fecal immunochemical test (FIT) kit by post. They were asked to return a stool sample via post in a sealed prepaid envelope.

Screening process

The primary screening modality used was the FIT, which detects hidden blood in the stool, a potential indicator of colorectal neoplasia. Participants who tested positive on the FIT were referred to the two-week wait cancer pathway for further diagnostic evaluation by colonoscopy, the gold standard for CRC diagnosis and prevention. Individuals with negative results were re-invited for routine screening at the recommended interval (usually every one to two years).

Endoscopic evaluation

Individuals referred for colonoscopy underwent a pre-procedure assessment, including medical history, medication review, and bowel preparation instructions. On the day of the procedure, bowel preparation agents (sodium picosulfate) were administered to ensure clear visualization of the colonic mucosa. Colonoscopy was performed by trained endoscopists using high-resolution video colonoscopes. Sedation was administered based on patient preference and the clinician’s choice. During the procedure, the entire colon was inspected, and any polyps or suspicious lesions were biopsied or removed using standard polypectomy techniques. Because EMR specimens can occasionally be fragmented, precise histological assessment of submucosal invasion depth (SM2 vs. SM3) may be more challenging; this limitation has been acknowledged in the manuscript. Specimens were sent for histopathological analysis. Tumor budding was not consistently reported in the pathology database over the study period and, therefore, could not be reliably extracted for analysis.

Follow-up

Surveillance-Based Approach

Individuals opting for a surveillance-based approach were followed up in the clinic at six months to assess for biochemical (serum carcinoembryonic antigen (CEA) levels) recurrence, followed by post-polypectomy colonoscopy and assessment for radiological (CT of the chest/abdomen/pelvis) recurrence at one year. If no further high-risk findings were noted, the next colonoscopy was at three years, with the patient then being potentially discharged if all was normal. In case of incomplete resection, margin involvement, or high-risk histology, the interval of colonoscopy was shortened (6-12 months).

Post-surgical Follow-Up

As with the surveillance-based approach, individuals who opted for prophylactic surgery were followed up in the clinic, initially at one month for clinical assessment, then at six months to determine biochemical (serum CEA levels) recurrence. CT of the chest/abdomen/pelvis for radiological recurrence was then done annually, for up to three years, to assess for radiological recurrence (especially node-positive cases). Furthermore, the first surveillance colonoscopy was at one year after surgery. If it was normal, the colonoscopy was repeated at three years, then every five years, depending on findings, age, and comorbidities.

Primary outcomes

The study evaluated the prognostic outcomes of patients with Kikuchi SM2 and SM3, focusing on the endoscopic recurrence, radiological recurrence, and biochemical recurrence in the surveillance-based group and the surgical excision group. The study also evaluated the cancer-related mortality in the two groups of patients. Both groups of patients were followed up at six-month and one-year intervals, with recurrence determined by the presence of tumor on colonoscopy, radiological evidence of recurrence on CT or MRI scans of the abdomen and pelvis, and CEA levels >0.5 ng/dL.

Secondary outcomes

Secondary outcomes focused on histological and clinical metrics, that is, the incidence of metastatic lymph node involvement in those undergoing surgery.

Statistical analysis

Data analysis was performed using SPSS Statistics for Windows, Version 24.0 (IBM Corp., Armonk, NY, USA). Descriptive statistics summarized baseline patient characteristics, presenting continuous variables as means with standard deviations (SDs) and categorical variables as percentages. Comparisons between patients who underwent surgery and those who chose a surveillance-based approach utilized the chi-square test for categorical variables. For categories where the expected frequency was less than 5, Fisher’s exact test was applied to ensure accuracy. A p-value of less than 0.05 was considered statistically significant for all analyses.

## Results

A total of 193 cases diagnosed with malignant colorectal polyps were reviewed, of which 70 were identified as sessile polyps with a Kikuchi classification of SM2 and SM3 and included in the study. The remaining 123 cases were excluded: 30 had a Kikuchi SM1 classification, and 93 were pedunculated polyps. Of the 70 cases included in the study, 23 patients underwent prophylactic surgical resection, while 47 patients opted for surveillance-based management. The study included patients ranging in age from 55 to 93 years, with a mean age of 76.19 years. No significant baseline differences were observed between the two groups in terms of age and gender. Table 1 presents the details of tumor characteristics and the baseline characteristics of the two groups.

**Table 1:** Tumor characteristics and baseline characteristics in the prophylactic surgery and surveillance-based groups.

	Prophylactic surgical resection	Surveillance-based approach
Tumor characteristics		
Most common location	Rectum	Rectum
Mean size of polyp	30.5 mm	25 mm
Most common histological type	Moderately differentiated adenocarcinoma	Moderately differentiated adenocarcinoma
Dysplasia	High grade	High grade
Baseline characteristics		
Age (median)	75 years	78 years
Gender (male:female)	26:21	13:10

When evaluating and comparing the prognostic outcomes between the two groups, we found that the degree of endoscopic and biochemical recurrence was significantly higher in the surveillance group compared to the surgical group. While the surgical management group had lower rates of radiological recurrence, these were not statistically significant. Additionally, cancer-related mortality was statistically significantly lower in the surgical group (Table 2).

**Table 2:** Comparison of oncological outcomes between the prophylactic surgical resection and surveillance-based groups. Test applied: chi-square test.

Prognostic outcomes	Prophylactic surgical resection	Surveillance-based approach	P-value	Chi-square value
Endoscopic recurrence	4	16	0.031	4.69
Radiological recurrence	4	6	0.65	0.206
Biochemical recurrence	3	18	0.031	4.619
Cancer-related mortality	0	1	0.019	5.488

Furthermore, it was noted that the lymph node involvement in patients undergoing prophylactic surgery was statistically significant (Table 3).

**Table 3:** Evidence of lymph node metastasis in individuals undergoing prophylactic surgical resection. Test applied: chi-square test.

Lymph nodes	Surgical management group
Evidence of metastasis	5
No evidence of metastasis	18
P-value	0.0048
Chi-square value	7.97

## Discussion

Kikuchi SM2 and SM3 classification implies intermediate to deep submucosal invasion, increasing the likelihood of lymph node metastasis and recurrence. According to Ichimasa et al. (2021), approximately 10% of T1 CRCs (with submucosal invasion) develop nodal metastases, necessitating further surgical management post-endoscopic resection [12]. Additionally, Son et al. (2022) confirmed that SM2-3 lesions substantially increase nodal metastasis risk in malignant polyps [13].

In our cohort of patients with Kikuchi SM2 and SM3 polyps identified via the UK’s national Bowel Cancer Screening Programme, prophylactic surgical resection was associated with significantly lower endoscopic and biochemical recurrence, and reduced cancer-related mortality, compared to surveillance. Specifically, endoscopic recurrence occurred in 4 surgical versus 16 surveillance cases (p = 0.031, chi-square = 4.69), biochemical recurrence in 3 versus 18 (p = 0.031, chi-square = 4.62), and cancer mortality was lower in the surgical group (p = 0.019, chi-square = 5.49). Moreover, lymph node metastases were found in 5 of 23 surgical patients (p = 0.0048, chi-square = 7.97), underscoring the diagnostic benefit of resection in staging.

These results support the hypothesis that Kikuchi SM2/3 lesions carry elevated metastatic and recurrence risk when managed conservatively. Surgical resection not only reduces recurrence but also provides critical pathologic staging insight that surveillance alone cannot. Collectively, these findings align with emerging evidence that SM2/3 lesions require careful risk stratification and, where feasible, surgical management.

Furthermore, we observed that the metastasis rate in the surgical group (~22%) was consistent with published data for deep invasion. With respect to the higher observed rate of tumor recurrence in the surgical cohort, this likely reflects selection bias, as patients referred for surgery often have additional high-risk features (e.g., adverse histology, incomplete endoscopic resection, or radiological concerns) that prompt operative management, predisposing this group to higher baseline recurrence risk. This concordance reinforces that SM2/3 lesions are high-risk, requiring more than surveillance. Therefore, surgery provides both oncologic control (by removing residual disease) and essential staging to guide adjuvant decisions.

Moreover, surveillance alone fails to detect occult nodal disease, which may contribute to recurrence and mortality. The survival benefit seen in the surgical group reinforces surgical excision as a management strategy for SM2/3 lesions in appropriate patients, especially in screening populations where early detection and treatment are possible.

The US Multi-Society Task Force on CRC (2020) recommended surgical resection when malignant polyps exhibit high-risk features, including deep submucosal invasion (SM ≥1 mm), positive margins, lymphovascular invasion, or poor differentiation. These guidelines endorse surgery when such features are present, reinforcing our findings that SM2/3 polyps carry a significant risk [1].

Beaton et al. (2013) provided evidence that deep submucosal invasion is a predictor of lymph node metastasis, underscoring the decision to pursue surgery when present [14]. Similarly, the meta-analysis by Vogel et al. (2022) reiterated that SM3 invasion is associated with a metastatic risk upwards of 22% and should influence resection decisions [15]. Our metastasis detection aligns precisely with this risk bracket.

Furthermore, in the study by Saraiva et al. (2021), low-risk malignant polyps, defined by superficial invasion, absence of lymphovascular invasion, and favorable differentiation, were safely managed without surgery, contrasting with higher-risk scenarios such as ours [16]. This contextualizes our divergence: SM2 and SM3, by definition, fall outside the low-risk criteria and appear to benefit more from surgery.

A dynamic predictive model by Liu et al. (2022) identified submucosal invasion depth, histologic grade, lymphovascular invasion, and other factors as independent predictors for lymph node metastasis, generating a nomogram with high discrimination (C-index ~0.91) [17]. This model underscores that SM2/3 is central to deciding whether additional resection is warranted.

Endoscopic recognition of deeply invasive lesions remains crucial. According to Mann et al. (2022), advanced imaging techniques (e.g., magnifying endoscopy, narrow-band imaging) and non-lifting signs help predict submucosal invasion before histology is available [18]. Identifying features such as depressed morphology or poor pit patterns facilitates early decision-making, potentially redirecting toward surgical management when SM2 and SM3 are suspected.

The Association of Coloproctologists of Great Britain and Ireland/British Society of Gastroenterology guidelines (2025) recommend multidisciplinary discussion for lesions with suspicious risk features, with a lower threshold to pursue surgery where deep invasion is likely [19]. This approach ensures that SM2/3 lesions, which often display visible high-risk features, are managed proactively.

Our findings reinforce that SM2 and SM3 polyps detected during screening should trigger multidisciplinary evaluation. Surgical resection offers both definitive histological staging and therapeutic removal of high-risk lesions. Given the 22% metastasis rate, surgery may improve long-term outcomes.

However, surgical morbidity, especially in older screening populations, remains a consideration. Shared decision-making should involve careful discussion of risks versus benefits, and guidelines from US, European, and Japanese societies all recommend tailoring management based on pathological risk and patient preference [20].

Patients with SM2/3 polyps but significant comorbidity or patient preference may elect surveillance if pathology demonstrates favorable additional features (e.g., no vascular invasion, well differentiation), though they should be counseled about potential residual risk. The threshold for surgery should be low where high-risk features are unequivocal.

Prospective multicenter studies are needed to validate outcomes for SM2/3 lesions managed surgically versus surveillance, with standardized histology, imaging, and follow-up protocols. Development of predictive nomograms incorporating histology and endoscopic features could personalize decisions, reducing overtreatment while maintaining oncologic safety. Collaboration across disciplines, including endoscopy, pathology, surgery, and patient representatives, is essential to refining guidelines grounded in high-quality contemporary evidence.

Strengths

This study has several key strengths, providing a focused and practical evaluation of Kikuchi SM2/3 polyps within the context of the UK Bowel Cancer Screening Programme. By directly comparing surgical resection with surveillance-based management, the study addresses a clinically significant and often debated decision point in early CRC care. The inclusion of multiple recurrence metrics, endoscopic, radiological, and biochemical, alongside cancer-related mortality, enhances the robustness of the outcome assessment. Importantly, the detection of lymph node metastases in the surgical cohort strengthens the case for resection, highlighting the diagnostic and prognostic value of formal staging. The retrospective design, while inherently limited, draws from a real-world screening population, increasing the applicability of the findings to routine clinical practice. Statistical comparisons, including chi-square analysis, were used to demonstrate meaningful differences between groups, particularly in recurrence and mortality outcomes, which consistently favored surgical intervention. The study’s focused cohort and clear definition of endpoints allow for a well-structured and relevant comparison that contributes timely evidence to support surgical management of SM2/3 lesions. Overall, this work strengthens the argument for early resection in selected patients and offers valuable insights for multidisciplinary decision-making in the management of screen-detected malignant polyps.

Limitations

The limitations of this study should be acknowledged when interpreting the findings. First, the retrospective design introduces inherent biases, including variability in case selection, treatment decisions, and documentation quality. Without randomization, it is possible that patients selected for surgery had additional clinical or histological risk factors that influenced decision-making, introducing selection bias. Second, the sample size was relatively small, particularly within the surgical cohort, which limits the statistical power and generalizability of the results. The assumption that surveillance patients had no lymph node metastases, due to lack of pathological staging, represents a potential data gap that could lead to an overestimation of the difference in metastatic rates. Additionally, follow-up duration was not uniform across patients and may not have been sufficient to capture all late recurrences or long-term outcomes. Variability in surveillance protocols and recurrence definitions (e.g., biochemical vs. radiological) could also impact the consistency of outcome reporting. Furthermore, as all lesions were resected using EMR, the potential for specimen fragmentation may limit the accuracy of submucosal invasion depth assessment (SM2 vs. SM3), and this should be considered when interpreting histological classification. Tumor budding, an emerging histopathological marker associated with lymph node metastasis, was not routinely reported during the study period and, therefore, could not be included in the analysis, representing an additional limitation. Lastly, as the study was conducted at a single center, institutional practices and endoscopic expertise may limit external applicability. Taken together, these methodological constraints, including retrospective data collection, sample size limitations, potential selection bias, and factors affecting pathological assessment, should be considered when interpreting the findings. Despite these limitations, the study offers important insights into the real-world management of SM2/3 lesions and highlights the need for prospective, standardized research in this area.

## Conclusions

For Kikuchi SM2/3 polyps identified via bowel screening, prophylactic surgical resection appears to offer superior oncologic outcomes, reducing recurrence risk, improving survival, and providing staging information, compared to surveillance. The detection of lymph node metastases in approximately 22% of surgical patients underscores the limitations of non-surgical management in this subgroup. While surgery bears procedural risks, its potential benefits warrant strong consideration when SM2/3 invasion is present. Surveillance may be appropriate only in carefully selected cases with additional low-risk features and patient-informed consent. Ongoing prospective research and improved imaging tools are needed to refine individualized management strategies going forward.
